# Recent Increase in Sex Ratio at Birth in Viet Nam

**DOI:** 10.1371/journal.pone.0004624

**Published:** 2009-02-27

**Authors:** Christophe Z. Guilmoto, Xuyên Hoàng, Toan Ngo Van

**Affiliations:** 1 CEPED (Université Paris Descartes Ined IRD), Paris, France; 2 General Statistics Office, Hanoi, Vietnam; 3 Hanoi Medical University, Hanoi, Vietnam; London School of Economics, United Kingdom

## Abstract

**Introduction:**

Since the 1980s, sex ratio at birth (male births per 100 female births) has increased in many Asian countries as a result of selective abortions, but to date there has been no such evidence for Viet Nam. Our aim in this paper is to ascertain the situation with respect to sex ratio at birth in Viet Nam over the past five years.

**Materials and Methods:**

Original data were obtained from sample population surveys in Viet Nam recording annual birth rates since 2000 of about 450,000 women, as well as from two successive birth surveys conducted for the first time in 2007 (1.1 million births). The annual population surveys include specific information on birth history and mothers' characteristics to be used for the analysis of trends and differentials in sex ratio at birth.

**Results and Discussion:**

Birth history statistics indicate that the SRB in Viet Nam has recorded a steady growth since 2001. Starting from a level probably close to the biological standard of 105, the SRB reached 108 in 2005 and 112 in 2006, a value significantly above the normal level. An independent confirmation of these results comes from the surveys of births in health facilities which yielded a SRB of 110 in 2006–07. High SRB is linked to various factors such as access to modern health care, number of prenatal visits, level of higher education and employment status, young age, province of residence and prenatal sex determination. These results suggest that prenatal sex determination followed by selective abortion has recently become more common in Viet Nam. This recent trend is a consequence of various factors such as preference for sons, declining fertility, easy access to abortion, economic development as well as the increased availability of ultrasonography facilities.

## Introduction

The rise in the sex ratio at birth (SRB, number of male births per 100 female births) has been evidenced in many Asian countries since the 1980s [1], [2]. It was first made visible by census results in China (1990 and 2000) [3] and in several Indian States (1991 and 2001 censuses, and surveys) [4], [5] as well as in South Korea [6]. The trend was detected more recently in other countries such as Georgia, Armenia or Azerbaijan where birth registration showed a rise in the masculinity of births over the course of the 1990s [7]. Other areas such as Singapore or Taiwan have been slightly affected, as well as Asian migrant populations living in Europe or North America [8], [9]. The recent rise in SRB across Asia is closely linked to the entrenched preference for sons, the decline in fertility levels and the spread of new sex determination technology. [2]

Viet Nam's SRB has also received a lot of attention for various reasons. To start with, Viet Nam shares more than a border with China: a lot of cultural communalities stem from their common history and shared cultural tradition, as illustrated by the impact of Confucianism, the patriarchal family system and the degree of son preference [10], [11]. Moreover, both countries have experienced a degree of social and economic change characterized by rapid economic development and a rapid decline in fertility accompanied by vigorous family planning campaigns by the Government and easy access to abortion facilities [12]. The main difference between the two countries is a gap of about 10 years in the onset of social, political and economic transformations in Viet Nam.

Beyond such comparison with China, late 20^th^ century's Viet Nam therefore presents many traits typical of countries where sex ratio at birth has risen above normal over the last 25 years. It has in particular undergone a rapid demographic transition over the last ten years. While child mortality rates have come down from 37 per 1000 to 16 in 1999–2006, fertility rates have recorded a fast decline in less than 20 years from 3.8 children per woman in 1989 to 2.1 in 2006 [13]. The population growth rates stands now at 1.3 percent and is poised to further decline in the future because of enduring low fertility.

However, so far interest in the sex ratio at birth in Viet Nam has to a large extent been frustrated by a lack of evidence. The 1999 census results on the sex ratio of the youth population and on the sex ratio of the birth prior to the census point to a possible slight bias towards male births with SRB values ranging from 105 to 108, even if the evidence has remained somewhat inconclusive according to the most detailed analysis [14]. Disaggregated data failed to reveal any pronounced regional bias against daughters in the 1990s [15]. Results of successive DHS survey conducted in 1997 and 2002 do not provide any further strong evidence of active sex selection [16], [17]. In fact, these two successive surveys yield conflicting estimates of both high and low sex ratio at birth for the 1988–1997 period, a discrepancy most likely due to the small size of the birth samples used for SRB calculations. In conclusion, while the possibility of a SRB level slightly higher than normal in 2000 cannot be ruled out, the absence of undeniable evidence and the uncertainty prevailing over available sources has led us to assume sex ratio at birth in Viet Nam to be close to expected biological values in the 1990s.

The lack of hard evidence from these sources is compounded by the absence of published civil registration statistics in the country. While surveys such as the MICS conducted in 2006 [18] attest to the reasonably good rate of birth registration across the country (88%), no data is available to monitor annual variations or regional differentials. A further deficiency is the almost complete absence of reliable abortion statistics in the country. The only available evidence pointing to the possibility of gender bias among Vietnamese parents is still indirect and limited to local data, small-scale surveys focusing on abortions and more qualitative field studies [14], [19]–[22].

The objective of this study is to collate the most recent information on the number of births by sex in the country and re-examine the possibility of a gradual demographic masculinization resulting from prenatal sex selection. To do this we will review newly available data collected by the Vietnamese General Statistics Office (GSO) and compute the recent trends in sex ratio at birth. We will also investigate some of its social and demographic correlates to help us understand the diffusion process at work. Other dimensions of gender discrimination among children such as sex specific mortality rates among children will not be examined here.

## Materials and Methods

Estimating SRB is made difficult by the sensitivity of this statistical indicator to sample size: estimates derived from a limited number of births from hospitals [23] or surveys [16], [17] may fluctuate within a large confidence interval. SRB therefore needs to be calculated from a large number of births. Misreporting is another potential issue, but we have no reason to believe that it plays a role in Viet Nam as significant as in China [24]. Furthermore, even series available from large countries with quality registration data always display minor year-to-year fluctuations that are partly attributable to random factors related to the number of annual births.

Two kinds of sources will be used in this paper and examined in turn. The principal source is the annual population survey conducted by the GSO from 2000 [13] These surveys are based on a 3% sample drawn from the 1999 Census. Information collected refers to households, general population as well as women of childbearing age. The sample is of considerable size, compared to other surveys such as the DHS. For the most recent survey conducted in March–April 2007, no less than 461,000 women aged 15–49 were surveyed. From this source, two types of data of interest to our analysis were collected. First, the surveys record the “last birth” born to all women aged 15–49. Using only births during the previous 12 months, we can thus estimate the sex ratio at birth during the previous year. But from 2006, the surveys have also included the birth history of all women of childbearing age by recording the year and sex of the last five live births and whether children are still alive. Using the raw data files, we will reconstruct the birth histories of mothers in the past from both the 2006 and the 2007 surveys and average them. The bias linked to missing mothers and mothers with more than six children appears limited. The survey also provides several variables including prenatal health care, prior knowledge of the sex of the foetus, social and economic characteristics of the women. After testing for significant differences with a chi-square statistic, we will identify a few variables characterizing subpopulations with significantly lower or higher SRB.

The main limitations of this source are of various types. On the one hand, the sample itself may still be too small for a detailed analysis of SRB differentials, such as regional variations among Vietnamese provinces, and may need updating as it is based on the 1999 census. Equally, available variables recorded in the survey are at times limited and of varying quality. In particular, data on the “last birth” by mothers tends to be biased towards sons in a population where women have often stopped bearing children after the birth of a son. The recently added retrospective question on the entire birth history of women corresponds precisely to the way in which to avoid this bias in the study of past variations in SRB.

In addition to its annual surveys, the General Statistics Office also initiated a special survey in 2007 across health facilities in the country to assess the number of births recorded in 2006. In terms of sample size, this survey is by far the best source to estimate sex ratio at birth as it is based on almost 1.1 million births in 2006 –amounting to about three quarters of the total number of expected births in the country. The survey was repeated the following year and preliminary results are also available. Results presented here have been corrected for the potential bias caused by the number of smaller, commune-level health centres that were omitted [25].

Compared to other available sources, this source has the unique advantage of allowing for the examination and mapping of spatial variations in SRB levels across the country. We will combine here the data from both surveys for 2006 and 2007. Since the number of births often remains limited when disaggregated for the 70-odd administrative provinces of Viet Nam, we have used a geostatistical technique to identify the clusters of high sex ratio in the country [26]. The technique is based on the computation of local indicators of spatial association (LISA), for which the statistical significance for regional clustering can be tested by randomized permutations using a technique developed by Luc Anselin [27]. Owing to Viet Nam's peculiar elongated shape, the procedure used here to test the statistical significance of this spatial clustering relies on first-order contiguity measurements rather than kilometric distance between provinces. This geostatistical method will allow us first to assess the level of spatial autocorrelation of SRB in Viet Nam and then to identify the cluster of high-SRB provinces found in the North of the country.

## Results

### Trends in SRB

**Figure 1:**
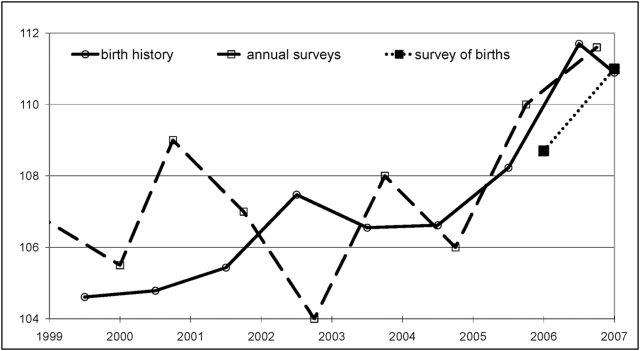
Sex ratio at birth in 1999–2007. Annual survey estimates are based on the births during the previous year and start with the initial sample data from the 1999 census. Birth history data are based on the averaged retrospective SRB values available from the last two population surveys. Estimates from the surveys of births are available only for 2006 and 2007.

We first examine the annual SRB estimates (Table 1, Figure 1). A first series is based on each survey, beginning with the 1999 census data (3% sample) up until the last survey conducted in 2007, and refers to the births over the previous 12 months. Data does not correspond exactly to the calendar year as annual sample surveys were usually conducted in April 1 or July 1 every year. A second series is prepared from the raw data files provided by the GSO. We have computed the retrospective annual SRB levels based on the birth history of mothers collected in 2006 and 2007, for which these data were available. Series from the 2006 and 2007 surveys were then averaged to yield a single annual series based on birth history.

**Table 1:** Estimates of sex ratio at birth in Viet Nam, 1998–2007.

1998	Sex ratio at birth			Size of birth samples			
Source	Annual surveys a		Survey of births c	Annual surveys a	2006 survey b	2007 survey b	Survey of births c
Method used	Last birth a	Birth history b		Last births	Birth history	Birth history	
**Year of birth**							
**1998**	107			39 733			
**1999**	105.5^a^	104.6		24 288	24 033	23 695	
**2000**	109	104.8		26 350	26 984	26 734	
**2001**	107	105.4		22 779	24 341	24 099	
**2002**	104	107.5		24 096	22 996	23 093	
**2003**	108	106.6		26 784	26 086	26 394	
**2004**	106	106.6		24 145	24 209	24 612	
**2005**	110	108.2		25 446	24 962	24 014	
**2006**	111.6	111.7	108.7	24 499	5 651^d^	24 536	1 478 082
**2007**		110.9	111.0			5 332^d^	1 443 526

a Estimates published in the annual *Surveys of Population Change*, *Labour Force and Family Planning* (General Statistics Office, Hanoi) except for 1999 (computed from the 2000 survey data).

b Computed by the first author from the 2006 and 2007 annual survey data files.

c Data for the survey of births in health centres conducted in 2007 and 2008 by the General Statistics Office and the Ministry of Health.

d Only available for the first trimester.

No clear trend seems visible from the last birth estimates up to year 2004: SRB values seem to fluctuate almost randomly within the 104–109 range. While this interval is above the normal sex ratio at birth (105–106), the difference is not however significant. Because of the limited size of the birth sample (about 25,000 births per year), the range of variations around 105–106 is in fact of ±3.5 and observed SRB values prior to 2004 in the successive surveys may be compatible with a normal SRB of 105–106. However, this series exceeded the 110 threshold in 2005. The latest SRB value for 2006 is 111.6 (±3.5), a level significantly higher than the biological standard, which corresponds to an active manipulation of the gender composition of the births.

The birth history data over the 1999–2006 period provides however a more precise picture of recent variations in SRB. While random fluctuations can be detected before 1999 (not shown here), the series is almost flat during the initial 1999–2001 period at a level close to 105. But after 2001, SRB values tend to increase gradually. The SRB starts at the level of 105 in 2001, reaches 108 in 2005 and 112 the following year. While the two series (births during the previous year and birth history) are similar for the most recent period, the SRB calculated using birth history yields a more robust estimate of the trend for the first years following the 1999 census. Obviously, the reconstruction of the entire birth history of mothers during the latest 2006 and 2007 annual surveys provides a more reliable picture of past SRB variations.

An independent confirmation of these results comes from the survey of births conducted in 2007 and 2008 across health facilities in Viet Nam. The SRB from this source –based as it is on a far larger sample– is 108.7 for births occurring in 2006. While it is possible that the sampling could be biased in favour of larger health facilities, this figure appears fairly consistent with the estimates derived from annual survey. Moreover, the confidence interval for this estimate is much smaller (108.7±0.4). The now available provisional SRB estimated from the subsequent survey of the 2007 births conducted in early 2008, based on 1 443 526 births, is 111.0 and confirms the continuous rise in SRB in Viet Nam. While it remains difficult to date the onset of the rise in SRB, its magnitude is now unmistakeable.

### SRB differentials within Viet Nam

The examination of SRB differentials within Viet Nam is of crucial importance for understanding the ongoing process of masculinization in the country by identifying some of the characteristics of the couples resorting to sex selection as well as their geographical location. Differentials may also help to monitor the potential diffusion in the near future of sex selection among other groups and regions that have been so far unaffected by the trend of rising sex ratio at birth.

Further processing of the data from the annual surveys allows us to identify several demographic and other characteristics associated with normal or skewed SRB within the Vietnamese population (Table 2). To do this, we will restrict our analysis to the 78,500 births recorded in 2007 which occurred in the three years prior to the survey to identify features associated with low or high sex ratio at birth. The first observation relates to women who reported no prenatal care, a home delivery or a delivery without medical personnel amongst whom SRB is significantly lower than among other women. These variables closely correspond to poorer access to modern health facilities, which is itself dependent on a host of factors such as distance to infrastructure, geographical remoteness and low financial means. Table 2 further indicates that low SRB is observed among less educated women and women reporting homemaking as their occupation. We find here a broad positive relationship between education and SRB, as often found in other Asian countries. Conversely and as could be expected, women who knew the sex of the children had a high SRB. Contraception users also have significantly more sons than the remainder of the sample. Other factors for higher SRB are also apparent, such as young age and specific economic sector such as collective sector and foreign company.

**Table 2:** Sex ratio at birth in various subpopulations, Viet Nam, 2006 and 2007 population surveys.

	Survey year	SRB
**Less than 5 years of education**	2007	101.8*
**Home work**	2006	104.5**
**No prenatal care**	2007	99.0**
**Delivery at home**	2007	101.9**
**Delivery with no medical attendance**	2007	103.2*
**Second birth**	2007	105.8**
**Prior knowledge of the sex**	2007	111.1**
**Contraception users**	2007	112.2**
**Aged less than 25 years**	2007	113.1**
**More than 4 prenatal visits**	2007	109.4**
**Work for government or a foreign company**	2006	114.4**

Data from the 2006 or 2007 surveys pertain to the three years before the survey.

Statistical significance of the chi-square test: * p<.10 **p<.05.

All these characteristics correspond to younger, more educated women who closely monitor their fertility and who are often from a more privileged background. However, it is worth mentioning that some of the usual correlates of high SRB such as urban residence, absence of male birth or high parity, do not appear to be significant in Viet Nam.

**Figure 2:**
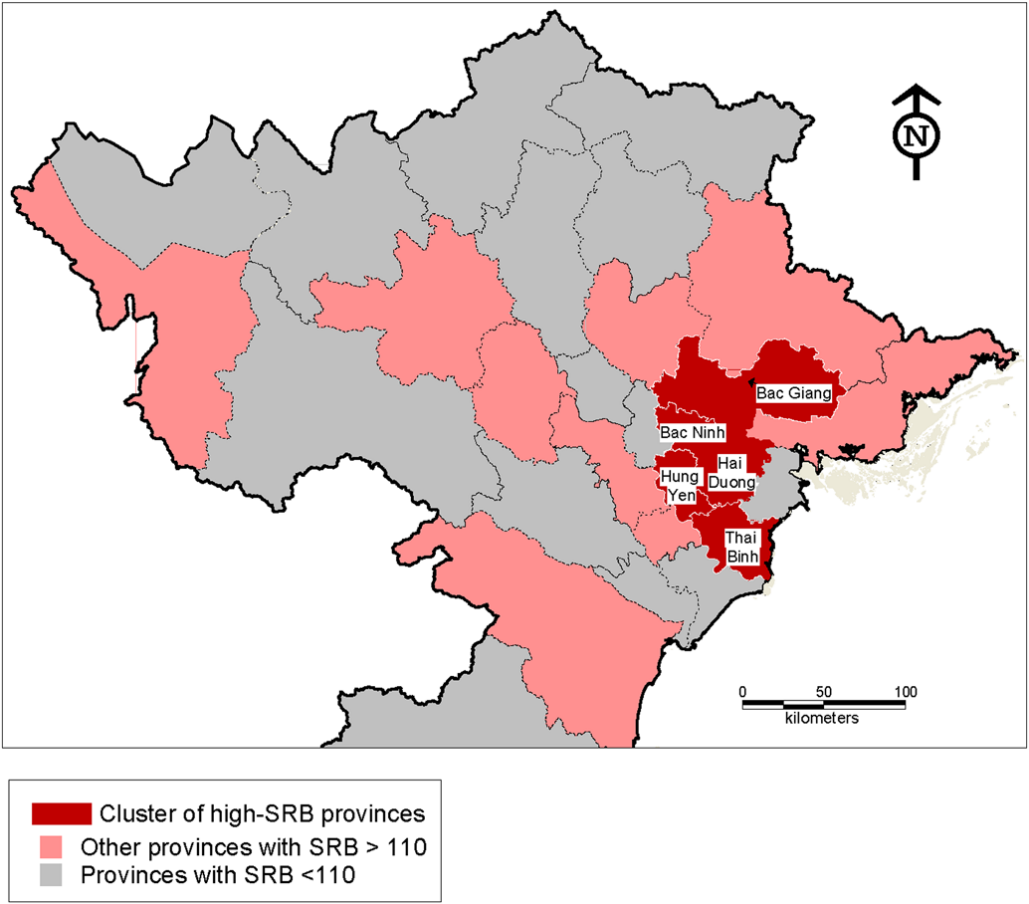
Regional cluster of high sex ratio at birth in 2006–2007 in North Viet Nam. The high-SRB regional cluster consists of provinces with SRB higher than average that are also surrounded by provinces with high SRB. The clustering's geostatistical significance is tested with the local indicator of spatial association (9999 permutations and p<.01).

Another feature systematically observed in Asia is the spatial patterning of skewed SRBs as seen in Northwest India or Eastern China [2], [28]. Many province-level estimates from the 2006 and 2007 birth surveys point in fact to SRB values clearly above 110. Using the geostatistical techniques described in the previous methodology section, we can indeed observe that Viet Nam's regional data from the two subsequent surveys of births display a very significant level of spatial autocorrelation in 2006–07 (Moran's I = 0.29): this indicates that contiguous provinces tend to share similar SRB levels. Provincial data that are mapped on Figure 2 are limited to the only regional cluster that is significant (p>.01) when testing with local indicators of spatial autocorrelation. This “hot spot” of skewed SRB encompasses the five provinces of Bac Giang, Bac Ninh, Hai Duong, Hung Yen and Thai Binh –with estimated SRB ranging from 112 to 121. This predominantly rural cluster is located between Hanoi and Hai Phong cities, at the heart of the Red River Delta: it corresponds to a densely populated agricultural region, which is both part of historical core of the country and deeply influenced by the economic transformations in post-reform Viet Nam. It is also surrounded by several more provinces with SRB greater than 110, with the notable exception of Hanoi and Hai Phong cities where SRB is below 110.

## Discussion

Our analysis establishes for the first time that SRB has increased over the past 5 years in Viet Nam. Compared to other countries, this increase appears both tardy and rapid. It undoubtedly came at a late stage compared to other affected countries from the Caucasus to South Korea, not to mention neighbouring China where the onset of gender imbalances dates back to the early 1980s. But at the same time, the rise in SRB seems to have proceeded quickly: starting from 105 in 2001, it exceeds 111 after 6 years, which corresponds to a rate of one SRB point per year. This rate of SRB increase in Viet Nam is higher than that measured in South Korea and China during the 1980s, but comparable to what observed Caucasian countries such as Azerbaijan or Armenia during the 1990s [2], [6], [7]. Even allowing for SRB to be already above 105 in 2000, a possibility that can be ruled out in view of conflicting data, the recent rise appears swift and steady. As a matter of fact, provisional results of the latest population survey in Viet Nam conducted in 2008 indicate a further SRB rise to 112 during the year preceding the survey.

As stated in the introduction, Viet Nam had almost all prior characteristics for an earlier rise in SRB –a patriarchal system and staunch son preference, demographic and economic change, strong family planning regulations and easy access to abortion– to which can be added a thriving private health sector since the late 1990s and a socially and culturally rather homogeneous country in which the diffusion of innovation can proceed rapidly. Skewed sex ratio at birth can be more specifically attributed to the conjunction of three components: biased gender preference linked to patriarchal family systems, low fertility encouraging people to resort to abortions (to avoid daughter) instead of having additional births (to bear a son), and available sex selection technology. We will now examine how the last two preconditions –low fertility and modern selection technology– may have played a determining role in the recent rise of sex ratio at birth.

Fertility decline in Viet Nam was incomplete in the early 1990s when the average number of children per woman was still of 3.3 [25], [29]. In fact, once contraception had spread across the country, the easiest way for women to ensure the desired gender composition was by additional child-bearing till the birth of a son, a practise corresponding to a male-biased stopping rule. As a result, the sex ratio of the last child tended to be severely biased towards boys as women often stopped having children after a son. But fertility continued to decline very fast during the 1990s to reach 2.3 children per woman by 2000 while stricter population regulations were also introduced in the country at end of the 1980s [30]. The strategy based only on repeated child-bearing became gradually inadequate and risky. The probability that couples with two children remain sonless is now of almost 25% and the “cost” associated to an additional birth is much higher than in the previous high-fertility system. Couples need therefore to plan in advance and more specifically to avoid unwanted female births. The need for sex selection has therefore been exacerbated by the continuous fertility decline observed during the 1990s. This new demand for sex selection technology has been first felt among sections of the population with the strong preference for sons and lowest birth rates and our previous analysis point for instance to specific populations in North Viet Nam.

But very low fertility is not the only condition for sex selection in Asia. For instance, sex ratio at birth was already as high as 114 in 1991 in the Indian state of Haryana with a fertility level still well above 3 children per woman. In Viet Nam, the growing demand for sex selection has also been frustrated by existing infrastructures in the country during the 1990s. In fact, a recent qualitative study conducted in different settings shows that a lot of Vietnamese women still rely on various folk methods –ranging from specific food diets to appropriate coital positions– to ensure the birth of a son [22]. Such age-old techniques of sex selection are no doubt inefficient, but their presence today confirms indirectly the rather recent introduction of modern prenatal technology in the countryside. The recent *Viet Nam Health Report* indeed stresses recent progress over the past ten years in the quality and availability of medical equipment in the country [31]. To a large extent, this overall picture also applies to the specific case of ultrasound machines imported from various countries: not only has it been easier to import better equipment, but the quality/cost ratio has improved with portable or 3-D units and decreasing production costs. In Viet Nam there are now some joint-venture companies producing imagery equipment. Though detailed data are not available, medical imaging equipment for ultrasonography, CT scan or MRI existing in the 1990s appeared to have been on the whole both scarce and of poor quality (equipment imported second-hand).

Statistics of access to ultrasounds performed in government health facilities are available from the Yearbooks of the Ministry of Health [32]: they point to an exceptional ten-fold upsurge in the number of ultrasound tests from 1.0 million in 1998 to 3.7 million in 2002 and 10.8 million in 2007. While these figures do not pertain specifically to obstetric sonography and do not include tests taken in private health centres, they demonstrate the rapid rise in the use of ultrasonography in the country. Similarly, studies conducted among Vietnamese women have already confirmed the very recent advance of ultrasonography in Viet Nam and the quality of newly available equipment. Prior determination of the sex of the foetus and its later confirmation through further scans are a major objective of pregnant mothers and explain in part the large public enthusiasm observed for the new technology [33], [34]. When the annual Population Change, Labour Force and Family Planning Survey asked in 2006 for the first time mothers about prenatal sex diagnosis, 63.5% among them declared they knew in advance the sex of their child.

Rapid fertility decline during the 1990s undoubtedly created a new demographic environment in which proactive sex selection became necessary. But it may also be hypothesized that the “supply-side” of the sex selection scenario has been also responsible for the somewhat belated arrival of modern prenatal sex selection in the country. As a result of better access to modern technology and growing demand for reduced offspring, gender discrimination has now emerged as a distinct feature of the current demographic transformations in Viet Nam, allowing families to avoid unwanted female births. The magnitude of the deviations from normal SRB levels is already significant at 110 and even higher in several provinces. In spite of the fact that the government has already introduced guidelines and legislation to make sex selective abortions illegal, their impact on SRB appears to be growing rapidly. Deterioration may be also fuelled by the current pace of transformation of Vietnamese society [35] and its economy and by the gradual spread of sex selection away from the initial cluster identified here. Moreover, with fertility still above 2.5 children per woman in many provinces, the scope for further degradation across the country in SRB levels in the future is real unless attitudes towards gender roles change rapidly. The trend evidenced by our findings requires close monitoring in a country of more than 85 million inhabitants where any long-term gender imbalances at birth will necessarily impact the demographic and social balance of society.
